# Poldip2 mediates blood-brain barrier disruption in a model of sepsis-associated encephalopathy

**DOI:** 10.1186/s12974-019-1575-4

**Published:** 2019-11-28

**Authors:** Daniel S. Kikuchi, Ana Carolina P. Campos, Hongyan Qu, Steven J. Forrester, Rosana L. Pagano, Bernard Lassègue, Ruxana T. Sadikot, Kathy K. Griendling, Marina S. Hernandes

**Affiliations:** 1 Division of Cardiology, Department of Medicine, Emory University School of Medicine, 101 Woodruff Circle, 308 WMB, Atlanta, GA 30322 USA; 2 Division of Neuroscience, Hospital Sírio-Libanês, São Paulo, SP Brazil; 3 Division of Pulmonary and Critical Care, Department of Medicine, Emory University School of Medicine, Atlanta, GA USA

**Keywords:** Poldip2, Lipopolysaccharide, Blood-brain barrier, Cyclooxygenase-2, Sepsis-associated encephalopathy, Brain microvascular endothelial cells

## Abstract

**Background:**

Sepsis-associated encephalopathy (SAE), a diffuse cerebral dysfunction in the absence of direct CNS infection, is associated with increased rates of mortality and morbidity in patients with sepsis. Increased cytokine production and disruption of the blood-brain barrier (BBB) are implicated in the pathogenesis of SAE. The induction of pro-inflammatory mediators is driven, in part, by activation of NF-κΒ. Lipopolysaccharide (LPS), an endotoxin produced by gram-negative bacteria, potently activates NF-κΒ and its downstream targets, including cyclooxygenase-2 (Cox-2). Cox-2 catalyzes prostaglandin synthesis and in the brain prostaglandin, E2 is capable of inducing endothelial permeability. Depletion of polymerase δ-interacting protein 2 (Poldip2) has previously been reported to attenuate BBB disruption, possibly via regulation of NF-κΒ, in response to ischemic stroke. Here we investigated Poldip2 as a novel regulator of NF-κΒ/cyclooxygenase-2 signaling in an LPS model of SAE.

**Methods:**

Intraperitoneal injections of LPS (18 mg/kg) were used to induce BBB disruption in Poldip2^+/+^ and Poldip2^+/−^ mice. Changes in cerebral vascular permeability and the effect of meloxicam, a selective Cox-2 inhibitor, were assessed by Evans blue dye extravasation. Cerebral cortices of Poldip2^+/+^ and Poldip2^+/−^ mice were further evaluated by immunoblotting and ELISA. To investigate the role of endothelial Poldip2, immunofluorescence microscopy and immunoblotting were performed to study the effect of siPoldip2 on LPS-mediated NF-κΒ subunit p65 translocation and Cox-2 induction in rat brain microvascular endothelial cells. Finally, FITC-dextran transwell assay was used to assess the effect of siPoldip2 on LPS-induced endothelial permeability.

**Results:**

Heterozygous deletion of Poldip2 conferred protection against LPS-induced BBB permeability. Alterations in Poldip2^+/+^ BBB integrity were preceded by induction of Poldip2, p65, and Cox-2, which was not observed in Poldip2^+/−^ mice. Consistent with these findings, prostaglandin E2 levels were significantly elevated in Poldip2^+/+^ cerebral cortices compared to Poldip2^+/−^ cortices. Treatment with meloxicam attenuated LPS-induced BBB permeability in Poldip2^+/+^ mice, while having no significant effect in Poldip2^+/−^ mice. Moreover, silencing of Poldip2 in vitro blocked LPS-induced p65 nuclear translocation, Cox-2 expression, and endothelial permeability.

**Conclusions:**

These data suggest Poldip2 mediates LPS-induced BBB disruption by regulating NF-κΒ subunit p65 activation and Cox-2 and prostaglandin E2 induction. Consequently, targeted inhibition of Poldip2 may provide clinical benefit in the prevention of sepsis-induced BBB disruption.

**Electronic supplementary material:**

The online version of this article (10.1186/s12974-019-1575-4) contains supplementary material, which is available to authorized users.

## Background

Sepsis is a life-threatening, systemic inflammatory response to infection commonly observed in patients with bacteremia [1, 2]. The endotoxin lipopolysaccharide (LPS), a cell wall component of gram-negative bacteria, is a potent mediator of sepsis pathogenesis [1, 2]. In the lab, purified LPS has been extensively used in vitro and in vivo to model sepsis and its associated complications [3]. Sepsis-associated encephalopathy (SAE), which is a diffuse cerebral dysfunction in the absence of direct central nervous system infection, is a frequent sequela of sepsis [1, 2]. Up to 70% of sepsis patients [4] and approximately half of patients with bacteremia [2, 5] develop SAE. Clinically, SAE presents as a rapid decline in cognitive functions and is associated with increased mortality and morbidity [2, 6]. Long-term neurocognitive deficits in memory, learning, and behavior have been reported; however, treatment of SAE remains limited to the management of the underlying infection [2, 7, 8].

Under physiologic conditions, the blood-brain barrier (BBB), formed by the interaction of astrocytes, pericytes, and endothelial cells, protects the brain from circulating insults [3]. Several mechanisms for the pathophysiology of SAE have been proposed, including BBB disruption [2] which has been observed in patients with SAE [9]. In animal models of sepsis [10–13], LPS induces astrocyte activation [14, 15] and detachment of pericytes from the basal lamina [13]. In addition to the effects of sepsis on these constituents of the BBB, the endothelium itself appears to be compromised [16, 17]. In vitro studies suggest LPS downregulates and induces disorganization of tight junction proteins, such as ZO-1 and occludin, in brain microvascular endothelial cells [12, 18].

The signaling preceding sepsis-induced BBB disruption is mediated by inducible systemic- and brain-derived inflammatory factors, such as IL-6 and TNF-α [1]. In gram-negative sepsis, LPS induces degradation of IκΒ and activation of NF-κΒ [1]. When active, NF-κΒ translocates to the nucleus where it promotes transcription of inflammatory mediators, including cyclooxygenase-2 (Cox-2) [19]. Cox-2 is a critical mediator of arachidonic acid metabolism and promotes prostaglandin E2 (PGE2) synthesis [20]. Physiological doses of PGE2 have been reported to be sufficient to induce brain endothelial permeability in vitro [21]. Moreover, inhibition of Cox-2 during neuroinflammation induced by traumatic brain injury [22], cerebral ischemia [23], and TNF-α challenge [24] attenuates BBB disruption, possibly by reducing levels of PGE2. The regulation of sepsis-associated BBB permeability mediated by a Cox-dependent pathway, however, requires further study.

Polymerase δ-interacting protein 2 (Poldip2) is a multifunctional protein that has been implicated in numerous cellular processes, including DNA damage repair, mitochondrial dynamics, cell cycle progression, and extracellular matrix deposition [25]. Recently, our group reported that Poldip2 mediates BBB disruption following cerebral ischemia [26]. Loss of Poldip2 in vivo attenuates hypoxia-induced expression of TNF-α and IL-6 [26], suggesting Poldip2 may play a role in regulating of NF-κΒ activity. Consistent with these observations, Poldip2 depletion blocks IκΒ degradation in cultured astrocytes [26]. Based on these results, we hypothesized that Poldip2 mediates BBB permeability induced by LPS via regulation of NF-kB/Cox-2 signaling.

In this study, we report that Poldip2 is a novel regulator of NF-κΒ/Cox-2 signaling in a model of SAE. Poldip2 heterozygous mice exhibit reduced BBB permeability, as well as reduced cortical levels of NF-κΒ subunit p65, Cox-2, and PGE2, following LPS-induced sepsis. Our data further suggest that Poldip2 depletion in cultured rat brain microvascular endothelial cells attenuates LPS-induced endothelial permeability and Cox-2 expression through inhibition of p65 nuclear translocation. These results thus provide new insights into the mechanisms by which Poldip2 regulates BBB permeability and suggest Poldip2/Cox-2/PGE2 signaling may play an important role in brain vascular physiology.

## Methods

### Animals

For all experiments, male and female Poldip2 heterozygous mice and littermate controls aged 8–16 weeks old were randomly assigned to control and experimental groups. The number of female and male animals in each group was approximately similar. Poldip2 gene trap mice on a C56BL/6 background were produced by Texas A&M Institute for Genomic Medicine (College Station, TX). Poldip2 heterozygous mice, previously characterized by Sutliff et al. [27, 28], were studied here because homozygous deletion of Poldip2 is perinatal lethal. Mice were genotyped using a standard 3-primer PCR method. All experimental protocols and animal handling procedures were approved by the Institutional Animal Care and Use Committee at Emory University (protocol reference number: 201700864).

### Tissue preparation for immunoblotting and ELISA

Poldip2^+/+^ and Poldip2^+/−^ mice were randomly assigned to LPS and phosphate-buffered saline (PBS) groups. Animals in the experimental group received an intraperitoneal injection (IP) of LPS (18 mg/kg, InvivoGen; Cat No. tlrl-eblps) isolated from *Escherichia coli* O111:B4 diluted in sterile PBS, while the control group received an equal volume of PBS. Following 6 or 18 h LPS or PBS treatment, mice were sacrificed by cervical dislocation. Cerebral cortices were isolated and flash frozen in liquid nitrogen before storage at − 80 °C. To prepare samples for analysis, cortices were lysed in 300 μl of buffer containing 0.3 M NaCl, 0.2% SDS, 0.1 M Tris base, 1% Triton X-100, 10 μg/ml aprotinin, 10 μg/ml leupeptin, 1 mM PMSF, and Halt phosphatase inhibitor cocktail (Themofisher Scientific; Cat No. 78428). Samples were subsequently processed using a tissue homogenizer before sonication and centrifugation (15,000 rpm) at 4 °C for 30 min. Finally, supernatants were collected and examined by immunoblotting or enzyme-linked immunosorbent assay (ELISA).

### ELISA

Prostaglandin E2 (PGE2) was measured in tissue lysates (prepared as described above) after 18 h of treatment using a commercially available ELISA (Abcam; Cat No. 133021) per the manufacturer’s instructions. A 4-parameter logistic curve was fitted to a standard, and experimental values were interpolated using GraphPad Prism software (version 7.0b). PGE2 levels were normalized to total protein concentration obtained by Precision Red Advanced Protein Reagent assay (Cytoskeleton; Cat No. ADV02).

### Meloxicam administration

For in vivo experiments, meloxicam (Putney; ANADA #200–540) 5 mg/kg was administered via subcutaneous (SQ) injection 10 h after an initial injection at the start of experiments. Animals were sacrificed after a total of 18 h and meloxicam-treated animals were compared to saline controls. For in vitro experiments, rat brain microvascular endothelial cells (RBMVECs) were treated with 100 μM meloxicam for 3 h, as previously validated [29].

### Measurement of BBB permeability in vivo

To evaluate alterations in cerebral vascular permeability, Evans blue dye was used as a marker of albumin extravasation, as previously described [30]. Briefly, intravenous injections of 2% Evans blue dye solution in PBS (4 ml/kg; MP Biomedicals; Cat No. 151108) were administered 18 h after IP administration of 18 mg/kg LPS. Evans blue dye was allowed to circulate for 10 or 30 min. Animals were then perfused transcardially with PBS until fluid from the right atrium became colorless. Whole brains were isolated, and the dye was extracted with formamide overnight at 50 °C. Subsequently, whole brains were allowed to dry for 1 h at room temperature before being weighed. Formamide dye concentration was quantified spectrophotometrically at 611 nm and normalized to the dry weight of both hemispheres.

### Tissue immunofluorescence

Eighteen hours after administration of PBS or LPS, mice were deeply anesthetized and subjected to transcardiac perfusion with a buffered saline solution, followed by a fixative solution containing 4% paraformaldehyde (PFA) dissolved in 0.1 mol/l phosphate buffer (PB, pH 7.4). The brains were collected, post-fixed in PFA for 4 h, and transferred to a 30% sucrose solution for 72 h in PB to ensure cryoprotection. Coronal sections (30 μm) were obtained on dry ice using a sliding microtome. Sections were incubated for 16 h with anti-Cox-2 (Cell Signaling; Cat No. 12282S; 1:100), anti-Poldip2 (custom made by GenScript; 1:250), and anti-CD31 (PECAM-1 BD Biosciences; Cat No. 550274; 1:100) antibodies. Antibodies were diluted in PB containing 0.3% Triton X-100 and 0.05% normal donkey serum (Jackson ImmunoResearch). Following three washes of 10 min each with PB, sections were incubated for 2 h with secondary antibodies conjugated to specific fluorophores for detection. Secondary antibodies used are as follows: anti-rabbit Alexa Fluor 488 (Jackson ImmunoResearch; Cat No. 711-545-152; 1:200) for Cox-2, anti-goat Alexa Fluor 647 (Millipore; Cat No, 180SA6; 1:200) for Poldip2, and anti-rat Alexa Fluor 568 (Millipore; Cat No, A11077; 1:200) for PECAM-1. Samples were mounted in Immu-Mount mounting medium (Thermo-Scientific). Images were obtained using a Zeiss LSM 800 Airyscan Laser Scanning Confocal Microscope System using a 40× oil objective lens and Zeiss ZEN acquisition software. Controls with no primary antibody showed no fluorescence. Finally, Z stacks were converted into a 3D projection using Bitplane Imaris 6.4.2 and MP4 files were generated using VCL and MPEG Streamclip software.

### Cell culture

Primary RBMVECs (CellBiologics; Cat No. RA-6023) were cultured on dishes coated with 0.1% gelatin from bovine skin (Sigma; Cat No. G6650). Culture media was supplemented with 2% fetal bovine serum, endothelial cell growth factors, and antibiotics (CellBiologics; Cat No. M1266-Kit). Cells were used from passage 4–6. For all experiments, RBMVEC monolayers were treated with 1 μg/ml LPS (Sigma; Cat No. L2630) isolated from *E. coli* O111:B4. For experiments concluding in immunoblotting, cells were lysed in a buffer containing 50 mM HEPES, 50 mM NaCl, 5 mM EDTA, 10 μg/ml aprotinin, 10 μg/ml leupeptin, 1 mM PMSF, and Halt phosphatase inhibitor cocktail.

### Small interfering RNA

Confluent RBMVECs were transfected with rat siPoldip2 (sense 5′-GUCUAUUGGUGGCGAUACU[dT][dT]-3′ antisense 5′-AGUAUCGCCACCAAUAGAC[dT][dT]-3′; Sigma) or control siRNA (MISSION siRNA Universal Negative Control #1; Sigma; Cat No. SIC001). Cells were washed with Hank’s balanced salt solution (HBSS) and transfected with 100 nM of siRNA and Lipofectamine RNAiMAX Reagent (volume equal to double the volume of siRNA; Invitrogen; Cat No. 13778150) in Opti-MEM reduced serum media (Gibco; Cat No. 31985-070). After incubating for 12 h, Opti-MEM was replaced with complete culture medium for an additional 72 h until LPS treatment was performed. Gene silencing was confirmed by immunoblotting.

### Immunoblotting

Cell and tissue lysates (prepared as described above) were assayed by Precision Red Advanced Protein Reagent to assess total protein concentration. Equal quantities of protein were aliquoted, and samples were brought to equal volume in Laemmli buffer and water before boiling for 10 min at 100 °C. Samples were resolved in acrylamide gels and transferred to PVDF membranes with a 0.45-μm pore size (Milipore; Cat No. IPVH00010). PVDF membranes were blocked in 5% bovine serum albumin (BSA) for at least 1 h before overnight incubation with primary antibodies diluted in Tris-buffered saline with 0.1% tween (TBST). Primary antibodies used are as follows: anti-Cox2 polyclonal rabbit Ab (Abcam; Cat No. Ab52237; 1:1000), anti-NF-κΒ p65 monoclonal rabbit Ab (Cell Signaling; Cat No. D14E12; 1:2000), anti-Poldip2 monoclonal rabbit Ab (Abcam; Cat No. Ab181841; 1:2000)**,** and anti-tubulin polyclonal rabbit Ab (Abcam; Cat No. Ab6046; 1:5000). Next, membranes were washed with TBST for 1 h before incubation with secondary antibody for an additional hour. Anti-rabbit horseradish peroxidase-conjugated secondary antibody (Cell Signaling; Cat No. 7074S) was used at half the concentration of primary antibodies. Bands were visualized using enhanced chemiluminescence (ThermoFisher; Cat No. 34580) and detected with Amersham Hyperfilm ECL (GE).

### Cell fractionation

RBMVECs were cultured, transfected with Poldip2 or control siRNA, and treated with LPS for 1 h as described above, and nuclear factions were isolated. Fractionation was performed as previously described [31]. Lamin B1 was used as a nuclear loading control and cytosolic contamination of nuclear fractions was assessed by blotting for β-tubulin.

### Measurement of endothelial permeability in vitro

HTS Transwell-24 well permeable support system with a 0.4-μm pore size (Corning; Cat No. 3396) was used to assess RBMVEC permeability induced by LPS. RBMVECs were transfected with siRNA against Poldip2 or control siRNA. The following day, 200,000 transfected cells were seeded on insert membranes that had been preincubated with complete culture medium for 2 h at 37 °C. Complete media was refreshed daily and after 48 h, cells were treated with 1 μg/ml LPS for 3 h at 37 °C. Alternatively, for in vitro permeability experiments involving meloxicam, 200,000 RBMVECs/insert were seeded and cultured for 72 h before simultaneous treatment with 1 μg/ml LPS and 100 μM meloxicam. Following treatment, transwell inserts were gently rinsed with warm HBSS and then placed in a 24-well plate containing 600 μl of HBSS per well. Next, 200 μl of 1 mg/ml fluorescein isothiocyanate (FITC)-dextran (molecular weight 4000; Sigma; Cat No. 46944) was added to the upper chamber and allowed to incubate at 37 °C for 15 min. Finally, the intensity of the dye in the lower chamber was spectrophotometrically assessed in a clear bottom black-walled microplate at 485 nm excitation and 520 nm emission.

### In vitro immunofluorescence

RBMVECs were seeded and cultured on gelatin-coated glass coverslips until confluent. Confluent monolayers were then transfected with Poldip2 or control siRNA as described above. Forty-eight hours post-transfection, cells were stimulated with 1 μg/mL LPS for 1 h. After 1 h, cells were washed twice in HBSS and fixed in 3.7% paraformaldehyde diluted in HBSS for 10 min at room temperature. Cells were then washed three times in PBS and permeabilized in PBS containing 0.2% Triton X-100 for 5 min followed by three washes in PBS for 2 min each. Blocking was performed in 0.5% BSA in PBS for 30 min. Anti-NF-κΒ p65 monoclonal rabbit Ab (Cell Signaling; Cat No. D14E12) was diluted 1:500 in blocking buffer and coverslips were incubated overnight at 4 °C with gentle rocking. The following day, coverslips were rinsed in blocking buffer (three washes × 5 min each) before incubation with secondary (Alexa488) antibody (1:500 dilution, Invitrogen) and DAPI (1:1000 dilution) for 1 h. Cells were then rinsed three times in PBS for 5 min each. Finally, coverslips were mounted on glass slides using Fluoromount-G (SouthernBiotech; Cat No. 0100-01).

### p65/DAPI colocalization analysis

Colocalization analysis was conducted using the ImageJ colocalization finder plugin. Images were acquired on a Zeiss LSM 800 confocal laser scanning microscope using a 63× oil immersion objective with blue (405 nM, DAPI) and green (488 nM, p65) channels. The Zen blue software application was used to acquire and save images. A minimum of five representative pictures (15–30 cells per picture) per condition were analyzed for each independent experiment. A total of four independent experiments were conducted. The Colocalization Finder ImageJ plugin was used to identify overlap between DAPI and p65 staining. Images displaying colocalized pixels were then measured for total area coverage and normalized to total DAPI area coverage. Results are presented as the mean fold change relative to control ± SEM. Representative figures are presented as the merged combination of DAPI (blue), p65 (green), and colocalization (white).

### Data analysis and statistics

Data are presented as mean ± standard error of the mean (SEM). Significance was determined by an unpaired *t* test for single comparisons and two-way analysis of variance (ANOVA) followed by Tukey’s post hoc test for multiple comparisons. A threshold of *p* < 0.05 was considered significant. All statistics were done using Prism 7 (GraphPad) software. For all assays concluding in immunoblot, films were scanned, densitometry was performed using ImageJ, and values were normalized to a loading control. For FITC-dextran transwell experiments, results are expressed as mean fold change relative to control.

## Results

### Heterozygous deletion of Poldip2 protects against LPS-induced BBB permeability

**Fig. 1:**
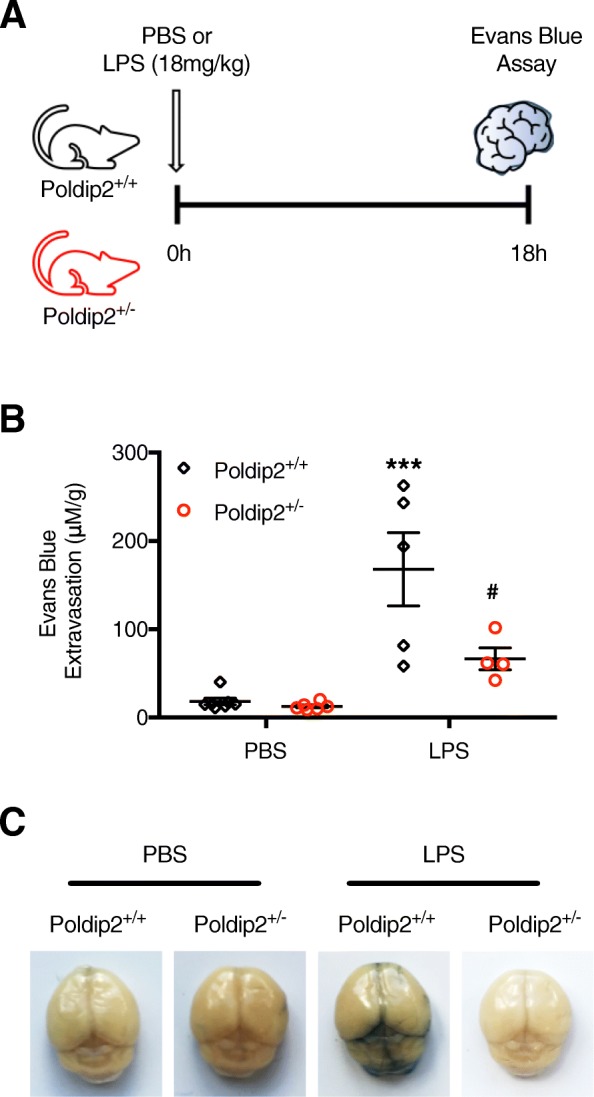
Heterozygous deletion of Poldip2 protects against LPS-induced BBB permeability. **a** Schematic of the experimental design. BBB disruption following 18 h LPS (18 mg/kg, IP) or PBS treatment was assessed by Evans blue dye extravasation in Poldip2^+/+^ and Poldip2^+/−^ mice. Evans blue dye was administered by intravenous injection and allowed to circulate for 30 min before animals were sacrificed. **b** The graph depicts Evans blue dye concentration extracted from whole brains normalized to dry brain weight. Bars represent mean ± SEM. Two-way ANOVA ***p < 0.001 vs. Poldip2^+/+^ + PBS, ^#^*p* < 0.05 vs. Poldip2^+/+^ + LPS, *n* = 4–7 mice/group. **c** Representative images of whole brains following Evans blue dye extravasation

We previously reported that Poldip2 mediates breakdown of the BBB following ischemic injury [26]. To test the hypothesis that Poldip2 also mediates BBB disruption in other instances of neuroinflammation, such as sepsis, Evans blue dye extravasation was used to assess changes in vascular permeability. Evans blue extravasation into whole brains was measured in Poldip2^+/+^ and Poldip2^+/−^ mice after 18 h of LPS treatment (Fig. 1a) since we previously reported that there is a 57% mortality rate among Poldip2^+/+^ animals after 20 h of LPS treatment (18 mg/kg) [32]. The large increase in Evans blue extravasation in Poldip2^+/+^ mice was significantly reduced in Poldip2^+/−^ mice (Fig. 1b, c). No difference in between female and male animals was observed (data not shown).

### LPS induces Poldip2 expression in the brain

**Fig. 2:**
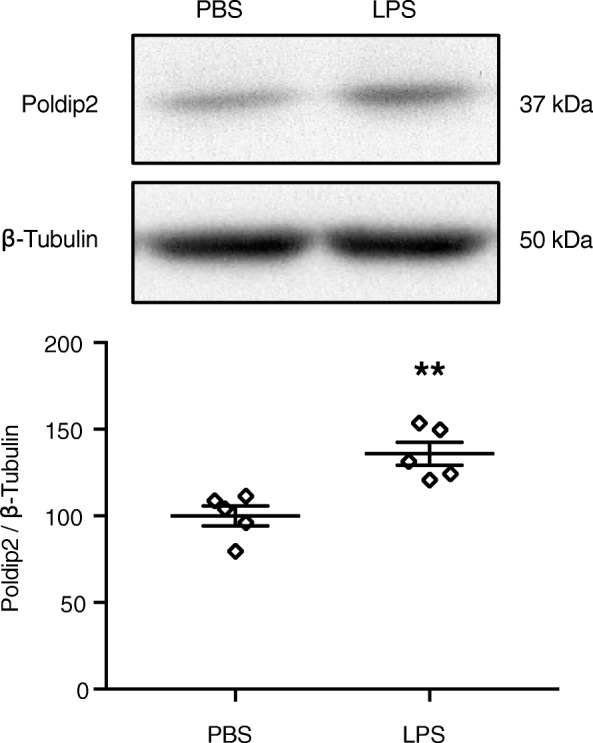
LPS induces Poldip2 expression in the brain*.* Poldip2 expression was measured in Poldip2^+/+^ cerebral cortices after 6 h of LPS (18 mg/kg, IP) or PBS treatment. Immunoblotting for Poldip2 was performed and β-tubulin was used as a loading control. Representative blots are shown. The graph depicts Poldip2 expression normalized to β-tubulin. Bars represent mean ± SEM. Unpaired *t* test, **p < 0.01 vs. PBS, *n* = 5 mice/group

Given the effect of Poldip2 on LPS-induced BBB permeability, we next sought to investigate the effect of LPS on Poldip2 expression. Very little is known about the regulation of Poldip2 [25]; however, Poldip2 expression was modestly, but significantly, induced in the cerebral cortices of Poldip2^+/+^ after 6 h of LPS treatment (Fig. 2).

### Heterozygous deletion of Poldip2 abrogates LPS-induced NF-κΒ/Cox2 signaling in vivo

**Fig. 3:**
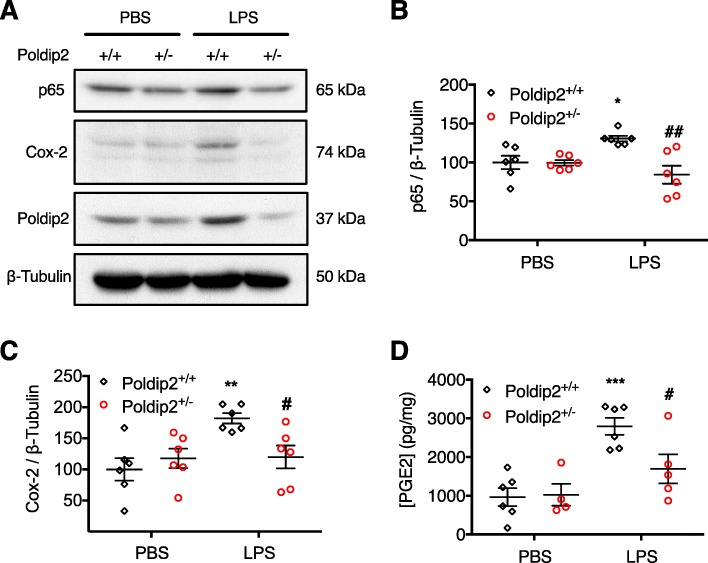
Heterozygous deletion of Poldip2 abrogates LPS-induced NF-κΒ/Cox2 signaling in vivo*.* Poldip2^+/+^ and Poldip2^+/−^ mice were treated with LPS (18 mg/kg, IP) or PBS to examine NF-κΒ/Cox2 signaling in vivo. **a** Immunoblotting for the NF-κΒ subunit p65 and Cox2 was performed on cerebral cortices of Poldip2^+/+^ and Poldip2^+/−^ mice after 6 h of treatment. β-tubulin was used as a loading control. Representative blots are shown. **b** p65 expression was quantified by densitometry. The graph depicts p65 expression normalized to β-tubulin. Error bars represent mean ± SEM. Two-way ANOVA *p < 0.05 vs. Poldip2^+/+^ + PBS, ^##^*p* < 0.01 vs. Poldip2^+/+^ + LPS, *n* = 6 mice/group. **c** Cox-2 expression was quantified by densitometry. The graph depicts Cox-2 expression normalized to β-tubulin. Bars represent mean ± SEM. Two-way ANOVA **p < 0.01 vs. Poldip2^+/+^ + PBS, ^#^*p* < 0.05 vs. Poldip2^+/+^ + LPS, *n* = 6 mice/group. **d** PGE2 levels in the cerebral cortices of Poldip2^+/+^ and Poldip2^+/−^ mice were assessed by ELISA after 18 h of LPS or PBS. The graph depicts PGE2 concentrations normalized by total protein concentration. Bars represent mean ± SEM. Two-way ANOVA, ***p < 0.001 vs. Poldip2^+/+^ + PBS, ^#^*p* < 0.05 vs. Poldip2^+/+^ + LPS, *n* = 4–6 mice/group

Opening of the BBB in SAE is mediated by inducible proinflammatory factors [6]. In gram-negative sepsis, LPS induces degradation of IκΒ, an inhibitor of NF-κΒ, and nuclear translocation of NF-κΒ [1, 33]. In the nucleus, NF-κΒ promotes transcription of proinflammatory mediators, including Cox-2 [19]. We previously reported that depletion of Poldip2 blocks IκΒ degradation in cultured astrocytes [26]. Thus, we sought to investigate the effects of Poldip2 depletion on LPS-induced NF-κΒ signaling in vivo. Poldip2^+/+^ and Poldip2^+/−^ mice were treated with LPS or PBS for 6 h and cerebral cortices were examined by immunoblotting (Fig. 3a). The NF-κΒ subunit p65 (Fig. 3b) and Cox-2 (Fig. 3c) were significantly upregulated in Poldip2^+/+^ cortices, which was not observed in Poldip2^+/−^ cortices. Likewise, PGE2, a major product of Cox-2 arachidonic acid metabolism, was upregulated in Poldip2^+/+^ cortices but not Poldip2^+/−^ cortices after 18 h of LPS (Fig. 3d).

### Poldip2 and Cox2 co-localize in brain endothelial cells

**Fig. 4:**
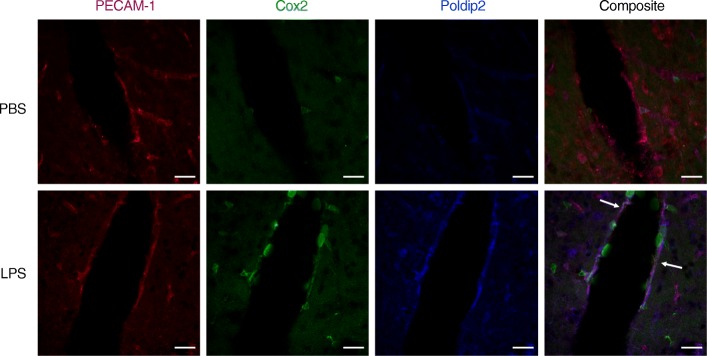
Poldip2 and Cox2 co-localize in brain endothelial cells. Immunofluorescence microscopy was used to examine Poldip2 and Cox-2 localization in vivo. Poldip2^+/+^ mice were treated with LPS (18 mg/kg, IP) or PBS for 18 h before brains were isolated and prepared for staining. Antibodies against PECAM-1 (red), Cox-2 (green), and Poldip2 (pseudo-colored in blue) were used. Composite image depicts overlay of Cox-2, Poldip2, and PECAM-1 images. Scale bars equal 20 μm. Representative images are shown. *n* = 3–4 mice/group

Endothelial cells are an integral component of the BBB and LPS treatment has been shown to induce endothelial permeability [12]. Thus, we next sought to examine the expression of Poldip2 and Cox-2 in brain endothelial cells following LPS treatment. Poldip2^+/+^ animals were treated with PBS or LPS for 18 h and brains were stained for Poldip2, Cox-2, and PECAM-1, which was used as an endothelial cell marker [34]. Poldip2 and Cox-2 were induced by LPS treatment, consistent with our previous results, and co-localized in endothelial cells (Fig. 4, Additional file 1: Video S1). Interestingly, while Cox-2 was also highly expressed in cells infiltrating from the capillary luminal space, these cells did not stain strongly for Poldip2.

### Poldip2 mediates LPS-induced NF-κΒ/Cox-2 signaling in vitro

**Fig. 5:**
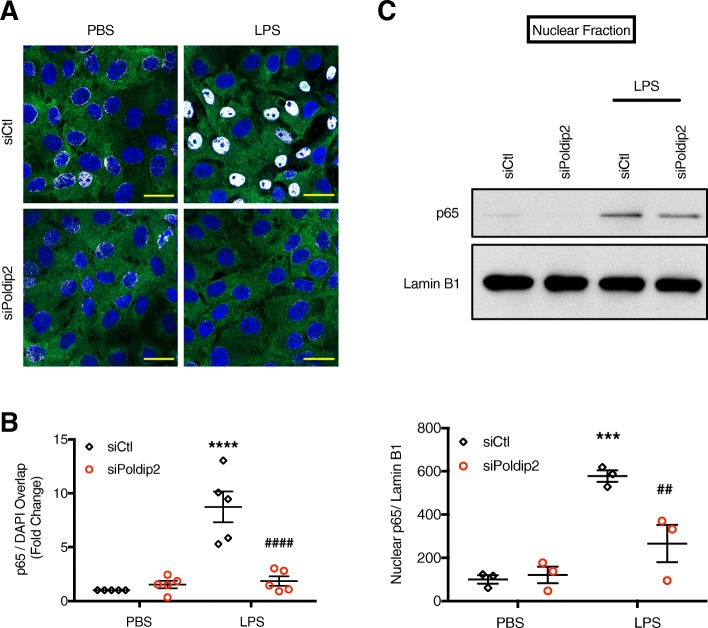
SiRNA against Poldip2 blocks LPS-induced p65 nuclear translocation. Confluent RBMVECs were transfected with siRNA against Poldip2 (siPoldip2) or control (siCtl) and treated with LPS (1 μg/mL) or PBS. **a** Following transfection and PBS or LPS treatment for 1 h, RBMVECs were fixed and incubated with antibodies against p65 (green) and DAPI (blue). White represents areas of colocalization. Scale bars equal 20 μm. Representative images are shown. **b** The areas of overlap between DAPI and p65 was measured by ImageJ. The graph depicts the average fold change in colocalization ± SEM. Bars represent mean ± SEM of 4 independent experiments. Two-way ANOVA ****p < 0.0001 vs. siCtl + PBS, ^####^*p* < 0.0001 vs. siCtl + LPS. **c** After transfection and 1 h of treatment, RBMVECs were fractionated and the nuclear fraction was blotted for p65 and Lamin B1. Representative blots are shown. **d** The graph depicts nuclear p65 normalized to Lamin B1. Bars represent mean ± SEM of three independent experiments. Two-way ANOVA, ***p < 0.001 vs. siCtl, ^##^*p* < 0.01 vs. siCtl + LPS

To examine the specific role of endothelial Poldip2 in regulating NF-κΒ/Cox-2 signaling, we moved to an in vitro system. RBMVECs were transfected with siRNA against Poldip2 or control and treated with LPS for 1 h. Cells were then stained for the p65 subunit of NF-κΒ (Fig. 5a) and evaluated by immunofluorescence microscopy. LPS-induced p65 translocation was significantly blocked by siPoldip2 (Fig. 5b). Corroborating the immunofluorescence data, results from nuclear fractionation of cultured RBMVECs also suggest that endothelial Poldip2 signaling mediates p65 nuclear translocation in cells treated with LPS (Fig. 5c).

**Fig. 6:**
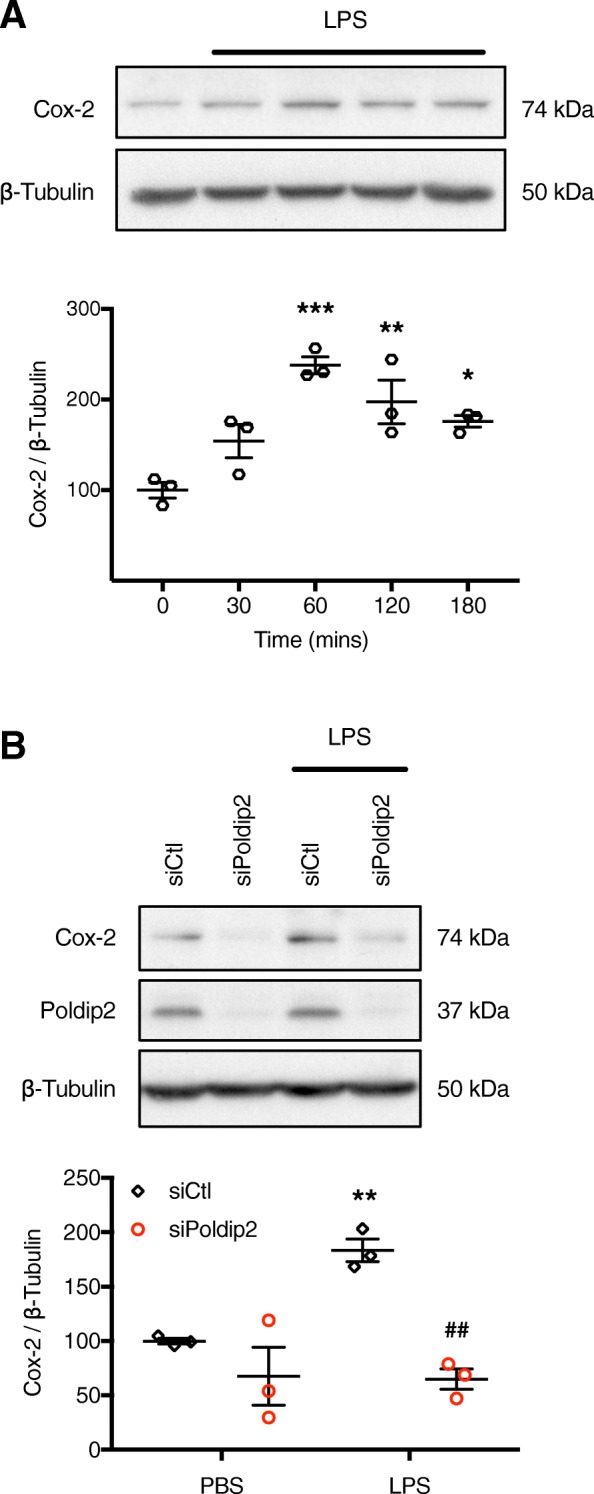
SiRNA against Poldip2 blocks LPS-induced Cox-2 induction. **a** Confluent monolayers of RBMVECs were treated with LPS for 0, 30, 60, 120, and 180 min before cells were lysed and immunoblotting for Cox-2 was performed. β-tubulin was used as a loading control. Representative blots are shown. The graph depicts Cox-2 expression normalized to β-tubulin. Bars represent mean ± SEM of three independent experiments. Two-way ANOVA, ***p < 0.001 vs. 0 min, **p < 0.01 vs. 0 min, *p < 0.05 vs. 0 min. **b** Cells transfected with siRNA against Poldip2 or control were treated with LPS or PBS for 1 h before cells were lysed and immunoblotting for Cox-2 was performed. β-tubulin was used as a loading control. Representative blots are shown. The graph depicts Cox-2 expression normalized to β-tubulin. Bars represent mean ± SEM of three independent experiments. Two-way ANOVA, **p < 0.01 vs. siCtl, ^##^*p* < 0.01 vs. siCtl + LPS

Subsequently, a time course for LPS-mediated Cox-2 induction was performed. Cox-2 was significantly induced after 1 h of LPS treatment (Fig. 6a). Using this timepoint, transfected RBMVECs were treated with LPS and Cox-2 expression was evaluated by immunoblotting. Consistent with the effect of Poldip2 depletion on p65 translocation, siRNA against Poldip2 significantly blocked LPS-mediated Cox-2 induction (Fig. 6b). Together, these results suggest that endothelial Poldip2 mediates activation of NF-κΒ and its downstream effectors by LPS.

### Poldip2 silencing and selective inhibition of Cox2 block LPS-induced endothelial permeability

**Fig. 7:**
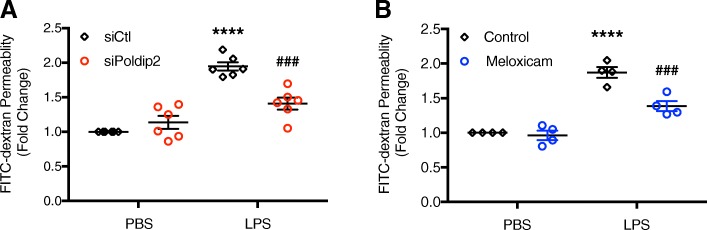
Poldip2 silencing and selective inhibition of Cox2 block LPS-induced endothelial permeability. RBMVECs were grown on transwell inserts and FITC-dextran diffusion into the lower chamber was quantified spectrophotometrically to assess changes in permeability. **a** RBMVECs were transfected with siPoldip2 or siCtl, seeded on transwell inserts, and treated with LPS (1 μg/mL) or PBS for 3 h before incubation with FITC-dextran. The graph depicts FITC-dextran concentration expressed as fold change relative to siCtl + PBS. Bars represent mean ± SEM of six independent experiments. Two-way ANOVA, ****p < 0.0001 vs. siCtl + PBS, ^###^*p* < 0.001 vs. siCtl + LPS. **b** RBMVECs were plated on transwell inserts and treated with LPS (1 μg/ml), meloxicam (10 μΜ), or both LPS and meloxicam simultaneously for 3 h before incubation with FITC-dextran. The graph depicts FITC-dextran concentration expressed as fold change relative to Ctl + PBS. Bars represent mean ± SEM of four independent experiments. Two-way ANOVA, ****p < 0.0001 vs. Ctl + PBS, ^###^*p* < 0.001 vs. Ctl + LPS

Inhibition of Cox-2 blocks BBB disruption induced by TNF-α [24], traumatic brain injury [22], and ischemic injury [23]. Based on these reports and the previously described effect of Poldip2 on BBB permeability [26], we next sought to investigate the role of Cox-2 and Poldip2 in LPS-induced endothelial permeability. RBMVEC permeability was assessed by FITC-dextran transwell assay after 3 h of LPS treatment. Consistent with our in vivo results, siPoldip2 significantly attenuated LPS-induced endothelial permeability (Fig. 7a). Similarly, selective inhibition of Cox-2 with meloxicam, significantly reduced FITC-dextran permeability in LPS-treated RBMVECs (Fig. 7b).

### Poldip2 mediates LPS-induced BBB permeability via Cox-2

**Fig. 8:**
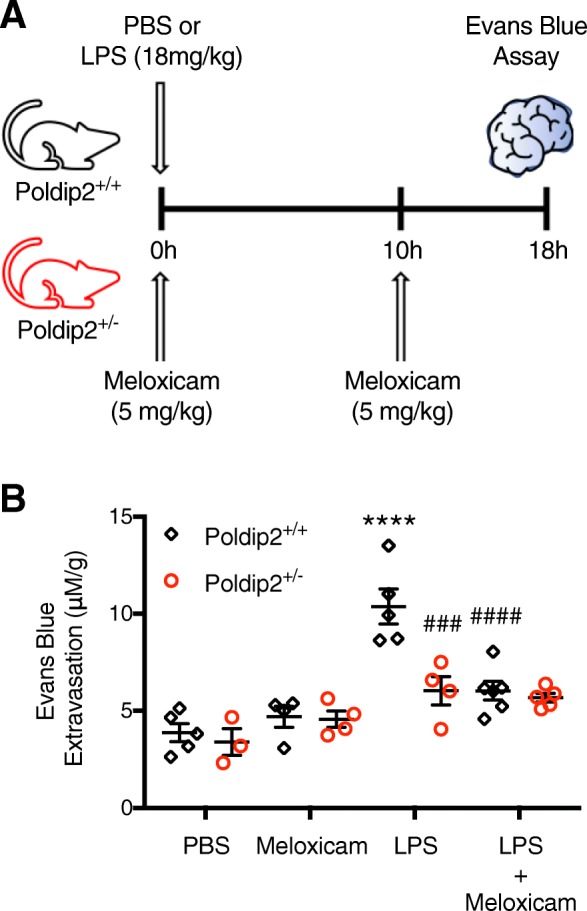
Poldip2 mediates LPS-induced BBB permeability via Cox-2. **a** Schematic of the experimental design. Evans blue dye was used to assess the effect of meloxicam on LPS-induced BBB permeability in Poldip2^+/+^ and Poldip2^+/−^ mice. Animals received PBS or LPS (18 mg/kg, IP) for 18 h, meloxicam (5 mg/kg) subcutaneously at time 0 and 10 h later, or both LPS and meloxicam. Evans blue dye was administered by intravenous injection and allowed to circulate for 10 min before animals were sacrificed. **b** The graph depicts Evans blue dye concentration extracted from whole brains normalized to dry brain weight. Bars represent mean ± SEM. Two-way ANOVA, ****p < 0.0001 vs. Poldip2^+/+^ + PBS, ^###^*p* < 0.001 vs. Poldip2^+/+^ + LPS, ^####^*p* < 0.0001 vs. Poldip2^+/+^ + LPS, *n* = 3–7 animals/group

Based on our findings that Poldip2 regulates NF-κΒ/Cox-2 signaling and mediates LPS-induced permeability, we sought to evaluate the role of Poldip2/Cox-2 signaling in vivo. Evans blue dye extravasation was used to assess BBB disruption in Poldip2^+/+^ and Poldip2^+/−^ mice after 18 h of PBS, meloxicam, LPS, or both LPS and meloxicam (Fig. 8a). Meloxicam alone had no significant effect on Evans blue extravasation in either Poldip2^+/+^ or Poldip2^+/−^ mice (Fig. 8b). Moreover, consistent with our previous results, LPS significantly induced Evans blue extravasation in Poldip2^+/+^ but not in Poldip2^+/−^ mice. Interestingly, meloxicam treatment blocked BBB disruption in LPS-treated Poldip2^+/+^ animals to the same extent as heterozygous deletion of Poldip2. Meloxicam had no significant effect in Poldip2^+/−^ mice treated with LPS. These results suggest Cox-2 is the primary mediator by which Poldip2 regulates LPS-induced BBB disruption.

## Discussion

In this study, we report a novel role for Poldip2 in LPS-induced SAE. Notably, heterozygous deletion of Poldip2 attenuated LPS-induced BBB permeability, NF-κΒ subunit p65 and Cox-2 expression, and subsequent PGE2 induction. Our in vitro results further suggest Poldip2 specifically regulates endothelial permeability, potentially via regulation of p65 translocation and Cox-2 expression.

The BBB, comprised of endothelial cells supported by astrocyte end-feet and pericytes, forms a selective barrier that maintains the microenvironment of the CNS [3]. Under pathological conditions, cytokines, matrix metalloproteinases, nitric oxide, purine nucleotides, and products of arachidonic acid metabolism promote endothelial permeability and a loss of BBB integrity [3]. BBB dysfunction exposes the CNS to systemic circulation, altering the extracellular milieu of the brain. These changes are associated with a number of neuroinflammatory and neurodegenerative disorders, including SAE [3].

Lipopolysaccharide, a cell wall component of gram-negative bacteria, activates NF-κΒ, which promotes transcription of inflammatory mediators, such as Cox-2 [1]. Consistent with previous reports, we found LPS-induced Cox-2 expression in the cerebral cortex of mice. Interestingly, PGE2, a downstream product of Cox-2, has been reported to be sufficient to induce brain microvascular endothelial permeability [21]. In line with a Cox-2/PGE2 model of BBB permeability, inhibition of Cox-2 during neuroinflammation induced by traumatic brain injury, cerebral ischemia, and TNF-α has previously been found to attenuate BBB disruption in vivo [22–24]. However, the intracellular signaling pathways leading to COX-2/PGE2 induction and permeability increases in the brain microvasculature are not well understood. In our study, heterozygous deletion of Poldip2 abrogated LPS-induced NF-κΒ expression with concomitant decreases in Cox-2 expression. While some of the Cox2 may be derived from endothelial cells, it is evident that infiltrating luminal cells, likely leukocytes, are major sources of Cox2 (Fig. 4, Additional file 1: Video S1). The fact that these cells express little Poldip2 suggests that loss of endothelial Poldip2 may either reduce leukocyte infiltration by promoting barrier integrity or reduce leukocyte Cox2 expression in a paracrine fashion. Either way, the protective effects of Poldip2 depletion on BBB integrity coincide with attenuated levels of LPS-induced PGE2 in Poldip2^+/−^ animals. Further implicating a Cox-2 dependent mechanism, meloxicam, a selective Cox-2 inhibitor, recapitulated the protective phenotype of Poldip2 depletion in LPS-treated Poldip2^+/+^ mice, while having no significant effect on LPS-treated Poldip2^+/−^ animals. While Poldip2 has previously been implicated in a range of cellular processes, including regulation of the proteasome [35] and reactive oxygen species (ROS) production [36], the role of Poldip2 in regulating effectors of arachidonic acid metabolism has not been previously been reported.

The regulation and cell specificity of Poldip2 remain poorly understood [25]. Here, we found that LPS modestly induced Poldip2 expression in cerebral cortices and staining of these cortices showed strong induction of Poldip2 in PECAM-1 labeled cells. As noted above, infiltrating leukocytes stain poorly for Poldip2, which may suggest differential mechanisms for Poldip2 regulation in endothelial cells and leukocytes. The effect of Poldip2 on leukocyte function is an area ripe for further investigation. Due to the integral role endothelial cells play in the BBB and the induction of Poldip2 in PECAM-1 labeled cells, together with the in vitro data that recapitulate the relationship between permeability, Poldip2 and Cox2 in cultured endothelial cells, we suggest that regulation of endothelial Poldip2 may be an important mechanism modulating Cox2 levels and BBB permeability either directly or indirectly. The protective effect of Poldip2 silencing in this LPS model of SAE is in agreement with our previous report that Poldip2 mediates BBB disruption following ischemic stroke [26].

In non-activated endothelial cells, NF-κB subunit p65 is trapped in the cytoplasm by inhibitory proteins such as IκΒ [1]. LPS stimulation induces IκΒ degradation, allowing NF-κΒ to migrate into the nucleus and bind DNA recognition sites in the regulatory regions of target genes [1]. In our study, Poldip2 depletion in RBMVECs blocked p65 nuclear translocation, providing a possible mechanism by which Poldip2 regulates LPS-induced Cox-2 expression. This finding is in agreement with our previous report that siPoldip2 blocks IκΒ degradation in cultured astrocytes. Together, these data strongly suggest Poldip2 is a novel NF-κΒ activator. The mechanism for this activation remains unreported; however, it may involve ROS, which can activate NF-κB. Poldip2 is known to enhance ROS production via its interaction with NADPH oxidase 4 in vascular smooth muscle cells [36]. Future research should focus on exploring this regulation.

## Conclusions

Poldip2 depletion blocks LPS-induced BBB permeability in a mouse model of sepsis-associated encephalopathy, likely through its effects on NF-κΒ/Cox-2/PGE-2 signaling. These results suggest Poldip2 represents a novel mechanism to regulate cerebrovascular permeability. Thus, Poldip2 may be an important therapeutic target in the prevention of sepsis-induced BBB permeability.

## Additional file

Additional file 1: **Video S1.** Poldip2 and Cox-2 co-localize in brain endothelial cells*.* Z-stacks of images from Fig. 4, depicting the cortices of Poldip2^+/+^ mice treated with LPS (18 mg/kg, IP) for 18 hours and subsequently stained for Poldip2 (pseudo-colored in blue), Cox-2 (green), and PECAM-1 (red), were converted into a 3D projection. Images are representative of three independent experiments.(MP4 17638 kb)

## Data Availability

All data supporting the conclusions of this study are included in this article.

## Abbreviations

ANOVA: Analysis of variance
BBB: Blood-brain barrier
BSA: Bovine serum albumin
CNS: Central nervous system
Cox-2: Cyclooxygenase-2
DAPI: 4′,6-diamidino-2-phenylindole
ELISA: Enzyme-linked immunosorbent assay
FITC: Fluorescein isothiocyanate
HBSS: Hanks’ balanced salt solution
IL-6: Interleukin-6
IP: Intraperitoneal
IκΒ: Inhibitor of NF-κΒ
LPS: Lipopolysaccharide
NF-κΒ: Nuclear factor kappa Β
PBS: Phosphate-buffered saline
PGE2: Prostaglandin E2
Poldip2: Polymerase (DNA-directed) delta-interacting protein 2
RBMVECs: Reactive oxygen species
ROS: Rat brain microvascular endothelial cells
SAE: Sepsis-associated encephalopathy
SEM: Standard error of the mean
siRNA: Small interfering RNA
SQ: Subcutaneous
TBST: Tris-buffered saline with 0.1% tween
TNF-α: Tumor necrosis factor alpha
